# Mitochondria as Therapeutic Targets in Heart Failure

**DOI:** 10.1007/s11897-022-00539-0

**Published:** 2022-02-11

**Authors:** Julia Schwemmlein, Christoph Maack, Edoardo Bertero

**Affiliations:** 1 Department of Translational Research, Comprehensive Heart Failure Center, University Clinic Würzburg, Würzburg, Germany; 2 Department of Internal Medicine and Specialties (Di.M.I.), University of Genoa, Genoa, Italy

**Keywords:** Mitochondria, Heart failure, Reactive oxygen species, MitoQ, Elamipretide, SGLT2 inhibitors, Cardiac metabolism

## Abstract

**Purpose of Review:**

We review therapeutic approaches aimed at restoring function of the failing heart by targeting mitochondrial reactive oxygen species (ROS), ion handling, and substrate utilization for adenosine triphosphate (ATP) production.

**Recent Findings:**

Mitochondria-targeted therapies have been tested in animal models of and humans with heart failure (HF). Cardiac benefits of sodium/glucose cotransporter 2 inhibitors might be partly explained by their effects on ion handling and metabolism of cardiac myocytes.

**Summary:**

The large energy requirements of the heart are met by oxidative phosphorylation in mitochondria, which is tightly regulated by the turnover of ATP that fuels cardiac contraction and relaxation. In heart failure (HF), this *mechano-energetic coupling* is disrupted, leading to bioenergetic mismatch and production of ROS that drive the progression of cardiac dysfunction. Furthermore, HF is accompanied by changes in substrate uptake and oxidation that are considered detrimental for mitochondrial oxidative metabolism and negatively affect cardiac efficiency. Mitochondria lie at the crossroads of metabolic and energetic dysfunction in HF and represent ideal therapeutic targets.

## Introduction

Cardiac contraction and relaxation are fuelled by the incessant conversion of energy afforded by mitochondrial oxidative metabolism. A remarkable feature of cardiac metabolism is its ability to swiftly increase the rate of substrate oxidation and adenosine triphosphate (ATP) synthesis in response to sudden elevations of ATP demand, such as those occurring during a bout of physical exercise. This ability relies on the tight coupling between the processes of contraction and relaxation and ion handling occurring in the cytosol and those of oxidative metabolism, mostly taking place in the mitochondrial matrix; this feature is denoted as *mechano-energetic coupling* [1].

**Fig. 1:**
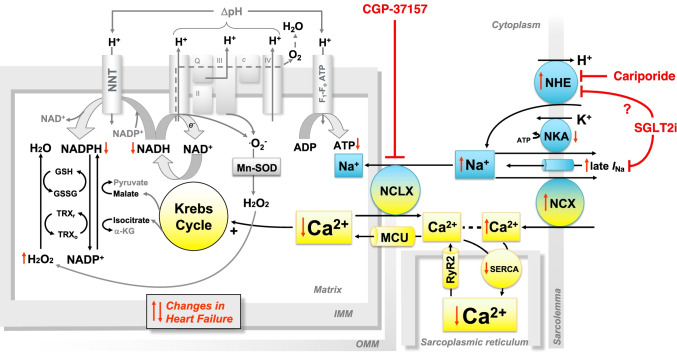
Drugs targeting mechano-energetic uncoupling in heart failure. In the normal heart, calcium (Ca^2+^) accumulation in the mitochondrial matrix stimulates the regeneration of reducing equivalents required for both adenosine triphosphate (ATP) production and hydrogen peroxide (H_2_O_2_) elimination. In the failing heart, decreased Ca^2+^ release from the sarcoplasmic reticulum and elevated cytosolic sodium (Na^+^) hinder mitochondrial Ca^2+^ accumulation, causing bioenergetic mismatch and oxidative stress. Drugs lowering cytosolic Na^+^ or inhibiting Ca^2+^ extrusion from mitochondria via the mitochondrial Na^+^/Ca^2+^ exchanger (NCLX) might ameliorate cardiac function by restoring mechano-energetic coupling. Other abbreviations: ADP, adenosine diphosphate; GSH/GSSG, reduced/oxidized form of glutathione; IMM, inner mitochondrial membrane; late *I*_Na_, late sodium current; MCU, mitochondrial Ca^2+^ uniporter; Mn-SOD, manganese-dependent superoxide dismutase; NAD^+^/NADH, oxidized/reduced form of nicotinamide dinucleotide; NCX, sarcolemmal Na^+^/Ca^2+^ exchanger; NKA, Na^+^/K^+^ ATPase; NNT, nicotinamide nucleotide transhydrogenase; OMM, outer mitochondrial membrane; RyR2, ryanodine receptor type 2; SERCA, SR Ca^2+^ ATPase; SGLT2i, Na^+^/glucose cotransporter 2 inhibitors; TRX_r_/TRX_o_, reduced/oxidized form of thioredoxin

Elevations of cardiac workload are accompanied by an increased turnover of ATP to adenosine diphosphate (ADP) in the cytosol. The increase in ATP demand needs to be matched by an increase in ADP phosphorylation, which is supported by a concomitant acceleration of the oxidative reactions of the Krebs cycle. This *parallel activation* is achieved by the accumulation of calcium (Ca^2+^) in the mitochondrial matrix, which stimulates the activity of Krebs cycle dehydrogenases. Elevations of heart rate and contractility are accompanied by an increase in the amplitude of cytosolic Ca^2+^ transients. As a result, more Ca^2+^ is driven in the mitochondrial matrix, where it boosts oxidative metabolism (Fig. 1).

The Ca^2+^-mediated stimulation of oxidative metabolism is relevant not only for ATP production, but also for mitochondrial antioxidant systems. In fact, reactive oxygen species (ROS) produced by oxidative phosphorylation are detoxified by enzymatic systems that are maintained in their reduced (i.e., active) form by reducing equivalents derived from the Krebs cycle. Specifically, the highly reactive superoxide (O_2_^−^) radical is rapidly converted to hydrogen peroxide (H_2_O_2_) by the manganese-dependent superoxide dismutase (Mn-SOD). In turn, H_2_O_2_ is reduced to H_2_O by peroxiredoxin (Prx) and glutathione peroxidases (Gpx), which are regenerated in their reduced (i.e., active) state by a cascade of redox reactions that ultimately require NADPH as a source of reducing equivalents (Fig. 1).

Alterations in cardiac energy metabolism and mechano-energetic coupling have been identified in virtually all stages and etiologies of heart failure (HF). In HF with reduced ejection fraction (HFrEF), derangements in cellular sodium (Na^+^) and Ca^2+^ handling impact both mitochondrial oxidative metabolism and ROS emission from the mitochondrial matrix [1]. In particular, reduction of sarcoplasmic reticulum (SR) Ca^2+^ load and elevation of cytosolic [Na^+^] in cardiac myocytes are hallmarks of HFrEF and conspire to hinder Ca^2+^ accumulation in the mitochondrial matrix. In fact, there is a close apposition between mitochondria and the SR, and the “privileged communication” between these two organelles enables efficient uptake of Ca^2+^ released from the SR inside the mitochondrial matrix via the mitochondrial Ca^2+^ uniporter (MCU) (Fig. 1). In HFrEF, decreased Ca^2+^ release from the SR hinders mitochondrial Ca^2+^ uptake [2••]. Furthermore, because Ca^2+^ is extruded from the mitochondrial matrix in exchange for Na^+^ imported from the cytosol, elevated [Na^+^] accelerates Ca^2+^ efflux from the mitochondrial matrix by increasing the driving force for Ca^2+^ extrusion [2••]. As a result, Ca^2+^ accumulation in the mitochondrial matrix is blunted in HFrEF, leading to insufficient regeneration of reduced NADH and NADPH required to support ATP production and H_2_O_2_ elimination, respectively [4.••••]. In addition, pathological elevations of workload deplete mitochondrial antioxidative capacity by regenerating reduced NADH from NADPH via the reverse-mode nicotinamide nucleotide transhydrogenase (NNT) reaction [5.••••]. Taken together, altered mechano-energetic coupling in HF compromises the regulation of mitochondrial oxidative metabolism, provoking an oxidation of the mitochondrial pyridine nucleotide pool that leads to bioenergetic mismatch and mitochondrial emission of ROS.

The therapeutic strategies based on our current understanding of mitochondrial dysfunction and mechano-energetic *uncoupling* in HFrEF, however, have not resulted in viable therapeutic options for HFrEF patients. In fact, antagonization of neuroendocrine activation remains the primary mode of action by which cardiac function, morbidity, and mortality can be improved in patients with HFrEF. Only recently, the clinical results of Na^+^/glucose cotransporter type 2 inhibitors (SGLT2i) have buttressed the concept that targeting metabolism holds great therapeutic potential. In this review, we discuss therapeutic approaches aimed at restoring function of the failing heart by targeting mitochondrial ROS, ion handling, and substrate utilization for ATP production.

## Targeting Mitochondrial Reactive Oxygen Species

Targeting ROS in cardiovascular diseases has been considered a promising therapeutic approach for a long time, but treatment with unspecific and untargeted antioxidants such as vitamin E failed to provide the anticipated results [6]. One potential explanation for the lack of efficacy of antioxidant therapies is the cellular compartmentalization of ROS, with mitochondrial—rather than general cellular—ROS formation and elimination playing a dominant role in the pathophysiology of HF and other cardiovascular disorders [7]. Mitochondrial ROS can be targeted by either using antioxidants that selectively accumulate in the mitochondrial matrix, such as mitoquinone (MitoQ), or preventing ROS production by stabilizing cardiolipin with agents such as the small peptide elamipretide.

### MitoQ

MitoQ consists of one ubiquinone moiety conjugated to the triphenylphosphonium cation, which drives its accumulation in the mitochondrial matrix [8, 9]. One caveat is that the antioxidative capacity of MitoQ is restored by electrons derived from the electron transport chain, and is therefore dependent on the Krebs cycle-mediated regeneration of reduced NADH; this might limit the antioxidative efficacy of MitoQ, and oxidized MitoQ has even been shown to possess pro-oxidative effects [10]. Preclinical studies demonstrated that ROS scavenging with MitoQ has cardioprotective effects in animal models of pressure overload–induced HF and ischemia–reperfusion injury [11, 12.•, 13, 14.•].

While MitoQ has not yet been tested in patients with HF, beneficial effects on vascular function were recently observed in humans. Dietary supplementation with MitoQ in healthy elderly improved endothelial function [15.•], and acute oral application had a positive impact on patients with peripheral artery disease, including improvement of flow-mediated vasodilation, walking capacity and time to claudication [16]. Oral treatment with MitoQ has also been clinically tested in other diseases associated with oxidative stress, such as chronic hepatitis C [17] or Parkinson’s disease [18].

### Elamipretide

Elamipretide (also known as SS-31, MTP-131 or Bendavia) is a cell-permeable small peptide selectively targeting cardiolipin [19, 20]. Cardiolipin is a phospholipid that is exclusively found in mitochondria, where it plays an important role in the structural and functional organization of the macromolecular complexes embedded in the inner mitochondrial membrane. For instance, cardiolipin stabilizes the respiratory chain complexes into supercomplexes for optimal oxidative phosphorylation [21]. The interaction between cardiolipin and cytochrome *c* determines whether the latter functions as an electron carrier or a peroxidase [20]. By stabilizing cardiolipin, elamipretide inhibits the peroxidase activity of the cytochrome *c*-cardiolipin complex, thus protecting its electron-carrying function. This optimizes mitochondrial electron transport and ATP synthesis and prevents ROS production at the electron transport chain [20, 22].

Preclinical studies with elamipretide yielded promising results, showing reduced infarct size and improved left ventricular (LV) contractile function in animal models of myocardial infarction and ischemia–reperfusion injury [23, 24, 25.•]. While these studies were mostly limited to rats, similar results were seen in other animal models including sheep, guinea pig, and rabbit [26, 27]. Furthermore, chronic treatment with elamipretide improved cardiac function in rodent [5.••, 28.••, 29] and canine models [30.•] of chronic HF.

Based on these promising preclinical results, elamipretide was tested in humans. In a double-blind, placebo-controlled, multicenter study in patients with ST elevation myocardial infarction and successful revascularization with percutaneous coronary intervention (PCI), intravenous treatment with elamipretide was associated with a small (~ 3%), non-significant reduction in infarct size, and a trend to a lower incidence of new-onset HF within the first 24 h after revascularization [31].

In one single-center trial in patients with HFrEF, a single 4-h infusion of elamipretide led to a significant dose-dependent improvement in LV function; however, results were limited by confidence intervals and missing correspondent changes in cardiac biomarkers [32]. A subsequent double-blind, randomized, placebo-controlled, multicenter trial assessing 70 participants with “stable” HFrEF receiving 4 weeks of subcutaneous treatment with elamipretide in addition to optimal medical therapy did not show significant changes in LV end-systolic volume (primary endpoint) or secondary outcomes [38.•].

Safety and efficacy of elamipretide were also assessed in patients with Barth syndrome (BTHS). BTHS is a rare inherited disease, caused by mutations in the gene encoding the mitochondrial transacylase tafazzin, which results in defective cardiolipin remodeling and consequent cardiolipin depletion [33, 34]. BTHS is characterized by cardiomyopathy, skeletal myopathy, neutropenia, and growth retardation [35]. Although subcutaneous treatment with elamipretide for 4 weeks did not affect primary or secondary endpoints in a first double-blind, placebo-controlled crossover study, in the 36-week open-label extension of the same trial, elamipretide significantly improved physical performance of BTHS patients [36.••].

In general, elamipretide was safe and well-tolerated in humans for intravenous [31, 32, 37] as well as subcutaneous application, the most common side effect being injection site reactions [36.••, 38.•, 39]. Assessment of elamipretide in clinical trials is still ongoing, also including patients with HF with preserved ejection fraction (HFpEF). In addition, a significant increase in LV stroke volume was observed in subjects with BTHS, although ejection fraction was not reduced at baseline in these patients [36.••].

Studies in explanted failing human hearts observed a protective effect of elamipretide on mitochondrial respiration [40]. Furthermore, the compound prevents cell death by inhibiting mitochondrial membrane permeability [5.••, 19], protects cristae networks from fragmentation [41, 42] and affects expression of genes related to mitochondrial energy metabolism [29, 43].

## Targeting Cellular Ca^2+^ Handling

Because mitochondrial antioxidant systems are supplied with reducing equivalents by NADPH derived from the oxidative reactions of the Krebs cycle, enhancing the Ca^2+^-mediated stimulation of the Krebs cycle dehydrogenases represents a viable strategy to prevent ROS emission from the mitochondrial matrix. In principle, this can be achieved by either augmenting mitochondrial Ca^2+^ uptake or by preventing Ca^2+^ extrusion via the mitochondrial Na^+^/Ca^2+^ exchanger (NCLX). While drugs that stimulate Ca^2+^ uptake via the MCU have not been tested in this context, preclinical studies showed that NCLX inhibition is an effective strategy to prevent oxidative damage.

### CGP-37157

CGP-37157 is a selective inhibitor of the NCLX, without relevant effects on L-type Ca^2+^ channels, sarcolemmal Na^+^/Ca^2+^ exchanger (NCX), or sarcolemmal Na^+^/K^+^-ATPase in cardiac myocytes [44, 45] (Fig. 1). In a guinea pig model of HF, NCLX inhibition with CGP-37157 elevated mitochondrial matrix Ca^2+^, shifting the redox state of mitochondrial NAD(P)H toward reduction and thus protecting cardiac myocytes from oxidative stress [4.••]. In vivo, treatment with CGP-37157 prevented maladaptive cardiac remodeling, LV dysfunction, and arrhythmias [46.••]. In addition, CGP-37157 counteracted the arrhythmogenic effects of glycosides ex vivo and in vivo. In fact, cardiac glycosides such as digoxin accelerate Ca^2+^ efflux via the NCLX by increasing cytosolic Na^+^, thus oxidizing the mitochondrial NAD(P)H pool and favoring ROS emission from the matrix [47]. Thus far, CGP-37157 has not been tested in humans.

### Cariporide

Because Ca^2+^ extrusion via the NCLX is driven by the Na^+^ gradient between the cytosol and the mitochondrial matrix, mitochondrial Ca^2+^ efflux can be counteracted by lowering cytosolic [Na^+^]. Cariporide reduces cytosolic [Na^+^] by selectively inhibiting the sarcolemmal sodium-hydrogen exchanger (NHE) type 1 (Fig. 1). NHE1 activation during myocardial ischemia leads to cytosolic Na^+^ and Ca^2+^ overload, consequently causing cardiac myocyte death. On these grounds, cariporide was clinically tested in the context of ischemia/reperfusion injury. Cariporide showed beneficial effects on systolic function and cardiac enzymes in a small pilot study conducted in patients with acute anterior myocardial infarction (MI) undergoing PCI [48]. However, in the subsequent GUARDIAN trial, which included a cohort of 11,590 patients with high risk of myocardial necrosis (including patients with unstable angina pectoris, non-ST elevation MI, or patients undergoing high-risk percutaneous or surgical revascularization), cariporide reduced the risk of death or MI exclusively in the subpopulation of patients undergoing bypass surgery, but not in the total study population [49]. Furthermore, the EXPEDITION trial focused on the efficacy and safety of cariporide in patients undergoing coronary artery bypass grafting; in this context, cariporide significantly reduced the risk of death or MI, but the effects resulted only from the decline in risk of MI, while mortality alone was slightly increased [50]. Therefore, although the largest clinical trial thus far demonstrated that treatment with cariporide is safe and well-tolerated [49], and despite proven benefits on myocardial ischemia/reperfusion injury, the increased risk of mortality in the EXPEDITION trial prevented cariporide from further clinical testing in cardiovascular trials [50].

### SGLT2 Inhibitors

The concept of targeting cytosolic Na^+^ has recently gained renewed interest in the light of the striking results of trials testing Na^+^/glucose cotransporter type 2 inhibitors (SGLT2i) in patients with HF with and without diabetes. SGLT2i lower circulating glucose levels by inhibiting its renal reabsorption via SGLT2 in the early proximal tubule [51]. Although these agents were developed as antidiabetic drugs, the attention on SGLT2i as a potential HF therapy increased after four large clinical trials assessing the cardiovascular safety profiles of three different SGLT2i in patients with diabetes mellitus and high cardiovascular risk demonstrated a substantial reduction of cardiovascular and all-cause mortality and a reduction in major adverse cardiac events [52–56].

Subsequent clinical trials focusing on patients with HFrEF confirmed that treatment with SGLT2i on top of optimal medical HF therapy reduces risk of cardiovascular and all-cause death as well as hospitalisations for HF. Subsequent clinical trials focusing on patients with HFrEF confirmed that treatment with SGLT2i on top of optimal medical HF therapy reduces risk of cardiovascular and all-cause death as well as hospitalisations for HF [57, 58]. Importantly, these results were independent of baseline diabetes status and were consistent across the continuum of glycated hemoglobin (HbA1c) levels, not differing between diabetes mellitus, prediabetes (HbA1c 5.7–6.4), or normoglycemia (< 5.7) [59.•, 60, 61]. Furthermore, in the EMPEROR-preserved trial, empagliflozin reduced the risk of hospitalization for HF in patients with HFpEF, regardless of the presence or absence of diabetes [60].

Since cardiac myocytes do not express SGLT2, and clinical trials used drug concentrations that do not inhibit SGLT1 [62], mechanisms of action explaining the cardioprotective effects of SGLT2i are currently under extensive investigation. Cardiac benefits might not be attributable to a single mechanism but rather result from several effects, including systemic ones such as natriuresis, lowering blood pressure, and preventing inflammation, as well as cardiac-specific effects including inhibition of NHE1 and changes in cardiac substrate preference.

A direct cardiac effect of empagliflozin was first proposed by the Baartscheer group, who observed that empagliflozin lowers cytosolic [Na^+^] by inhibiting the NHE1 in ventricular myocytes isolated from healthy rabbits and rats. The authors showed that, akin to the effect of cariproride, the Na^+^-lowering activity of empagliflozin results in reduced cytosolic [Ca^2+^] by favoring Ca^2+^ extrusion to the extracellular space via the NCX, and at the same time increases mitochondrial [Ca^2+^] by lowering the driving force for mitochondrial Ca^2+^ efflux via the NCLX [63.•]. The same group demonstrated that also canagliflozin and dapagliflozin inhibit NHE1 and reduce cytosolic [Na^+^] [64] (Fig. 1). NHE1 expression was confirmed in human atrial and ventricular tissue, and acute exposure to empagliflozin was able to significantly inhibit NHE1 activity in human atrial myocytes [62]. However, the concept of NHE1 inhibition has subsequently been challenged by Chung and colleagues, who could not reproduce the inhibitory effect of empagliflozin on NHE1, nor its Na^+^-lowering activity, by applying a variety of methods and a wide range of empagliflozin concentrations. The authors attributed the discrepancy to the unstable pH conditions of previous experiments. In addition, the general hypothesis of NHE1 inhibition was questioned, because NHE1 activity might be very low at physiological pH, in which case an inhibition would not result in any relevant effect [65.•].

NHE1 inhibition with SGLT2i is not limited to cardiac myocytes, but was also observed in human blood-derived myeloid angiogenetic cells and platelets (both cell types expressing NHE but not SGLT2). This might add to the systemic cardioprotective effects of SGLT2i by stabilizing atherosclerotic plaques and inhibiting thrombosis [66]. The precise interaction between SGLT2i and NHE1 is still unresolved. While a direct interaction of SGLT2i with the extracellular Na^+^-binding site of NHE was proposed [64], dapagliflozin was shown to enhance NHE1 gene expression by activating AMPK and increasing AMPK phosphorylation [67]. As discussed below, modulation of the AMPK pathway might also account for additional cardioprotective effects mediated by improvements in cellular and mitochondrial energetics.

In addition to their putative effects on NHE1, which are still controversial, SGLT2i were recently reported to inhibit the late sodium current (*I*_Na_) (Fig. 1) [68.••]. In fact, *I*_Na_ has long been considered the main mechanism to increase cytosolic [Na^+^] in cardiac myocytes of patients with HF [69–71]. Ranolazine inhibits *I*_Na_, and through lowering [Na^+^]_*i*_ improves diastolic function and has anti-arrhythmic effects *in vitro* [72–74] and in patients with atrial fibrillation when combined with dronedarone [75]. SGLT2i bind to Na_V_1.5 channels and inhibit *I*_Na_, and similar to tetrodotoxin, a classical Na_V_1.5 inhibitor, reduced inflammasome activation in an acute model of cardiac ischemia [68.••].

## Targeting Cardiac Metabolism

### Inhibitors of Fatty Acid Oxidation

**Fig. 2:**
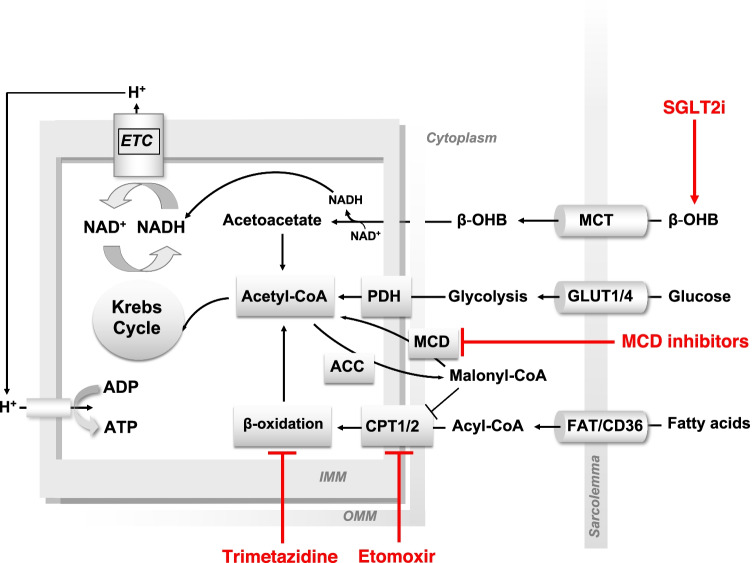
Drugs targeting substrate preference in heart failure. The normal heart relies primarily on glucose and fatty acid oxidation for adenosine triphosphate (ATP) production. Emerging evidence indicates that in the failing heart, oxidation of ketones such as β-hydroxybutyrate (β-OHB) might become a relevant source of ATP. Because ATP production from glucose or β-OHB oxidation requires less oxygen than fatty acid oxidation, a metabolic shift away from fatty acids toward glucose and/or ketone oxidation for ATP production increases cardiac efficiency. Inhibition of fatty acid β-oxidation can be achieved by (i) inhibiting carnitine palmitoyltransferase 1 (CPT1), which mediates fatty acid import in the mitochondrial matrix, with etomoxir; (ii) directly inhibiting β-oxidation with trimetazidine; (iii) increasing malonyl-CoA levels by inhibiting its degradation with malonyl-CoA decarboxylase (MCD) inhibitors. Furthermore, the hyperketonemic state associated with sodium/glucose cotransporter 2 inhibitors (SGLT2i) treatment may provide cardiac myocytes with a more energetically efficient substrate, i.e., β-OHB. Other abbreviations: ACC, acetyl-CoA carboxylase; ADP, adenosine diphosphate; ETC, electron transport chain; FAT/CD36, fatty acid translocase; GLUT1/4, glucose transporter 1/4; MCT, monocarboxylate transporter; NAD^+^/NADH, oxidized/reduced form of nicotinamide dinucleotide; PDH, pyruvate dehydrogenase

Whereas the healthy heart predominantly oxidizes fatty acids and glucose for ATP production, HF is characterized by changes in substrate utilization that vary depending on the etiology and stage of the disease [76]. Metabolic abnormalities in HFrEF include a progressive decline in myocardial phosphocreatine and, ultimately, also ATP levels, lending support to the widely accepted view that the failing heart is “an engine out of fuel” [77]. Because ATP production from fatty acid oxidation requires more O_2_ for each ATP compared with glucose, pharmacological agents inhibiting fatty acid oxidation and favoring more “O_2_-efficient” oxidation of glucose have been tested in animal models and patients with HF. These include etomoxir, trimetazidine (TMZ), and malonyl-CoA decarboxylase inhibitors (Fig. 2).

Etomoxir is an inhibitor of the mitochondrial carnitine palmitoyltransferase 1 (CPT1), the rate-limiting enzyme of fatty acid β-oxidation. CPT1 inhibition shifts cellular substrate preference away from fatty acids toward glucose [78]. Etomoxir was initially developed for the use in diabetes, but was also tested in patients with HF. While one initial study observed significant improvements in LV ejection fraction and cardiac output during exercise in 10 patients [79], a subsequent clinical trial in a larger cohort of patients with congestive HF had to be stopped early because of hepatotoxicity, since a small number of participants in the treatment group exhibited an abnormal increase in circulating liver enzymes [78]. Consequently, therapeutic use was not further pursued, even though the general concept was suggested to be beneficial.

Trimetazidine (TMZ) inhibits β-oxidation of free long-chain fatty acids by competitive inhibition of the long-chain 3-ketoacyl-CoA thiolase [80.•, 81], thus promoting glucose utilization for ATP production. By virtue of its O_2_-sparing activity, TMZ relieves angina symptoms, and is recommended by the current European Society of Cardiology (ESC) guidelines as a second-line treatment to reduce angina frequency in patients whose symptoms are not controlled with other antianginal medications [82]. However, in the large randomized controlled ATPCI trial, TMZ did not affect the recurrence of angina in patients who had either elective PCI for stable angina or urgent PCI for unstable angina or non-ST-segment elevation MI [83].

In patients with HF, treatment with TMZ increased the phosphocreatine/ATP ratio [84.••], which was associated with improvement of symptoms, exercise capacity, and overall quality of life. Furthermore, treatment with TMZ was shown to preserve LVEF and counteract maladaptive ventricular remodeling in HFrEF [84.••, 85–87] [reviewed in [86, 87] ]. While most of these studies focused on patients with ischemic HFrEF, TMZ improved physical exercise tolerance and cardiac function also in patients with idiopathic dilated cardiomyopathy with and without diabetes [88, 89]. However, these studies were limited by the small study population and the observational design; the impact of TMZ on symptoms and cardiovascular outcomes in HF has not been conclusively demonstrated in randomized controlled trials thus far.

Malonyl-CoA levels dictate the rate of fatty acid oxidation in mitochondria by inhibiting the import of fatty acyl-CoA in the mitochondrial matrix. Malonyl-CoA is derived from carboxylation of acetyl-CoA, which becomes more abundant in states of energy repletion, and is degraded by the malonyl-CoA decarboxylase (Fig. 2). Pharmacological inhibitors of malonyl-CoA decarboxylase provide an alternative approach to modulate fatty acid oxidation and favor glucose utilization. Inhibition of malonyl-CoA decarboxylase in a rat model of MI decreased cardiac fatty acid oxidation rates and prevented HF development [90]. Malonyl-CoA decarboxylase inhibitors have not been tested in humans thus far.

### Metabolic Effects of SGLT2 Inhibitors

The canonical mode of action of SGLT2-inhibitors (SGLT2i) is to inhibit glucose reabsorption in the proximal renal tubule, thereby inducing urinary glucose excretion. By lowering the total glucose pool, SGLT2i induce a fasting-like state characterized by increased lipolysis and ketogenesis [91], resulting in lower HbA1c levels and weight loss. Changes in systemic metabolism have an impact on myocardial substrate utilization and might contribute to the cardioprotective effect of SGLT2i by improving cardiac efficiency. It has been proposed that the increase in circulating ketone levels induced by SGLT2i is cardioprotective by providing the energy-starved failing heart with a “thrifty substrate” [92] (Fig. 2). In line with this hypothesis, SGLT2i ameliorated LV remodeling and enhanced cardiac efficiency in a swine model of MI. This was accompanied by a metabolic shift toward increased oxidation of ketones, fatty acids, and branched chain amino acids [93.••]. In a mouse model of HFpEF, SGLT2i increased cardiac ketones in a similar fashion as a ketone diet, attenuated NLPR3 inflammasome formation and antagonized proinflammatory cytokine-triggered mitochondrial dysfunction and fibrosis [94]. It is important to note that while SGLT2i increase circulating ketones in diabetic patients, their ketogenic effect is markedly lower in nondiabetic subjects [95, 96]. In addition, the failing heart exhibits an increased reliance on ketone oxidation for ATP production, and it is unclear whether further enhancing ketogenesis in HF has additional effects on this metabolic shift [97, 98].

Another intriguing hypothesis is that the fasting-like state induced by SGLT2i activates cellular sensors that respond to low-energy states, such as sirtuin 1 (SIRT1) and AMPK [99.•]. Both SIRT1 and AMPK are normally activated during nutrient deprivation and orchestrate cellular responses to starvation via post-translational modifications of proteins. Activation of SIRT1 and AMPK has pleiotropic effects that might explain the benefit of SGLT2i on cardiac and renal function; these include the activation of autophagy, i.e., the degradation of dysfunctional cellular components, including damaged mitochondria. This model is buttressed by numerous studies showing that autophagic removal of dysfunctional mitochondria is protective against cardiac stressors [100–102], but experimental evidence confirming that this process mediates the cardioprotective activity of SGLT2i is lacking.

## Conclusions

Mitochondria lie at the crossroads of metabolic and energetic dysfunction in HF and represent ideal therapeutic targets. Mechano-energetic uncoupling, characterized by a mismatch of energy supply and demand, results in energy depletion and oxidative stress as a common mechanism in HFrEF. While antagonization of neuroendocrine activation remains the cornerstone of HFrEF treatment, the recent clinical results of SGLT2i suggest that targeting metabolism holds great therapeutic potential. In this context, the concept that targeting mitochondrial redox regulation and ion handling may impact on cardiac metabolism and remodeling is an emerging field, and more research is needed to translate the knowledge from preclinical models to the clinical situation.
